# Asymtomatic Mass In The Tail Of The Pancreas: Intrapancreatic Accessory Spleen (IPAS)

**DOI:** 10.34172/aim.2022.91

**Published:** 2022-08-01

**Authors:** Fatma Yıldırım, Fazli Erdogan, Mehmet Kilic, Melike Rusen Metin

**Affiliations:** ^1^Department of Pathology, Ankara City Hospital, Ankara, Turkey; ^2^Department of Pathology, Ankara Yildirim Beyazit University, Ankara, Turkey; ^3^Department of General Surgery, Ankara Yildirim Beyazit University, Ankara, Turkey; ^4^Department of Radiology, Medipol University, Pendik Hospital, Istanbul, Turkey

**Keywords:** Accessory spleen, Intrapancreatic accessory spleen, Neuroendocrine tumors

## Abstract

We present a 48-year-old male patient with a mass in the tail of the pancreas on abdominal ultrasonography. The lesion was suspicious for a well-differentiated pancreatic neuroendocrine tumor and spleen preserved distal pancreatectomy surgery was performed. It was diagnosed as intrapancreatic accessory spleen (IPAS) after pathological examination. Accessory spleen is not an infrequent congenital entity caused by the localization of normal splenic tissue in ectopic regions. As it is known, an accessory spleen is a benign entity and does not require surgical treatment or follow-up when detected. However, it is important to recognize IPAS tissue as it may mimic a pancreatic neoplasia when it is located in the pancreas. In this article, we discuss the differential diagnostic possibilities of the IPAS entity.

## Introduction

 Accessory spleen is a common congenital abnormality caused by the localization of normal spleen tissue in ectopic regions. In autopsy studies involving the general population, the accessory spleen was found at a rate of 10%–15%.^1^ The most frequent location is the splenic hilum (80%), followed by the pancreas (17%), phrenicocolic ligament, adipose capsule of the kidney, mesentery, stomach, and jejunum.^2,3^ The patients are usually asymptomatic and it is diagnosed incidentally during imaging studies performed for other conditions. Accessory spleen is a benign condition, and does not require surgical treatment or follow-up. Nevertheless, when it is located within the pancreas, it can mimic a pancreatic neoplasm radiologically, particularly neuroendocrine tumors. In order to protect patients from unnecessary surgery, it is important to differentiate IPAS from pancreatic neoplasms.^4^

## Case Report

 We present a 48-years-old male patient who was investigated for an incidentally detected pancreatic mass. The patient, who was followed up for hypertension and diabetes mellitus, was admitted to our center after a hypoechoic mass was located in the pancreatic tail on the abdominal ultrasound performed for routine screening. Abdominal magnetic resonance imaging (MRI) revealed a 21 × 17 mm solid mass lesion with exophytic growth in the pancreas tail. On contrast and non-contrast magnetic resonance images, the solid mass was a hypervascular lesion with hypointense signal on T1 sequences and hyperintense signal on T2 sequences. Radiographically, the differential diagnosis included neuroendocrine tumors and low probability adenocarcinoma; therefore, histopathological confirmation was recommended. The patient underwent spleen-preserving distal pancreatectomy.

 Macroscopically, resection material containing 6.5 × 5 × 2.5 cm distal pancreas was examined. The gross pathological findings showed a 1.9 cm in diameter, dark red-brown, well-circumscribed, solid nodule that was surrounded by normal pancreatic tissue (Figure 1). Microscopically, the solid lesion was consistent with the usual spleen tissue and was reported as intrapancreatic accessory spleen (IPAS) tissue (Figure 2).

**Figure 1:**
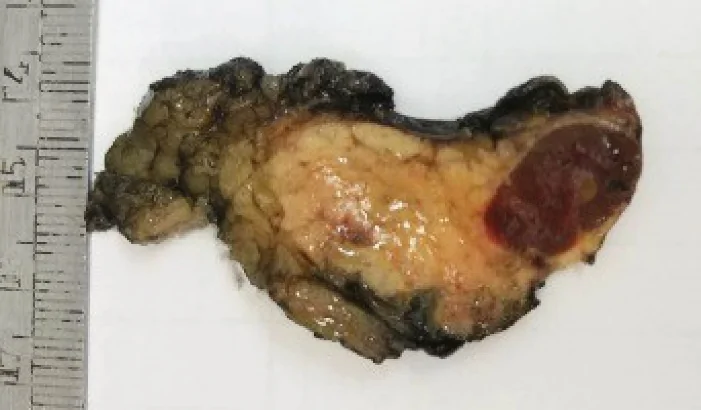
Macroscopic View of the Intrapancreatic Mass

**Figure 2:**
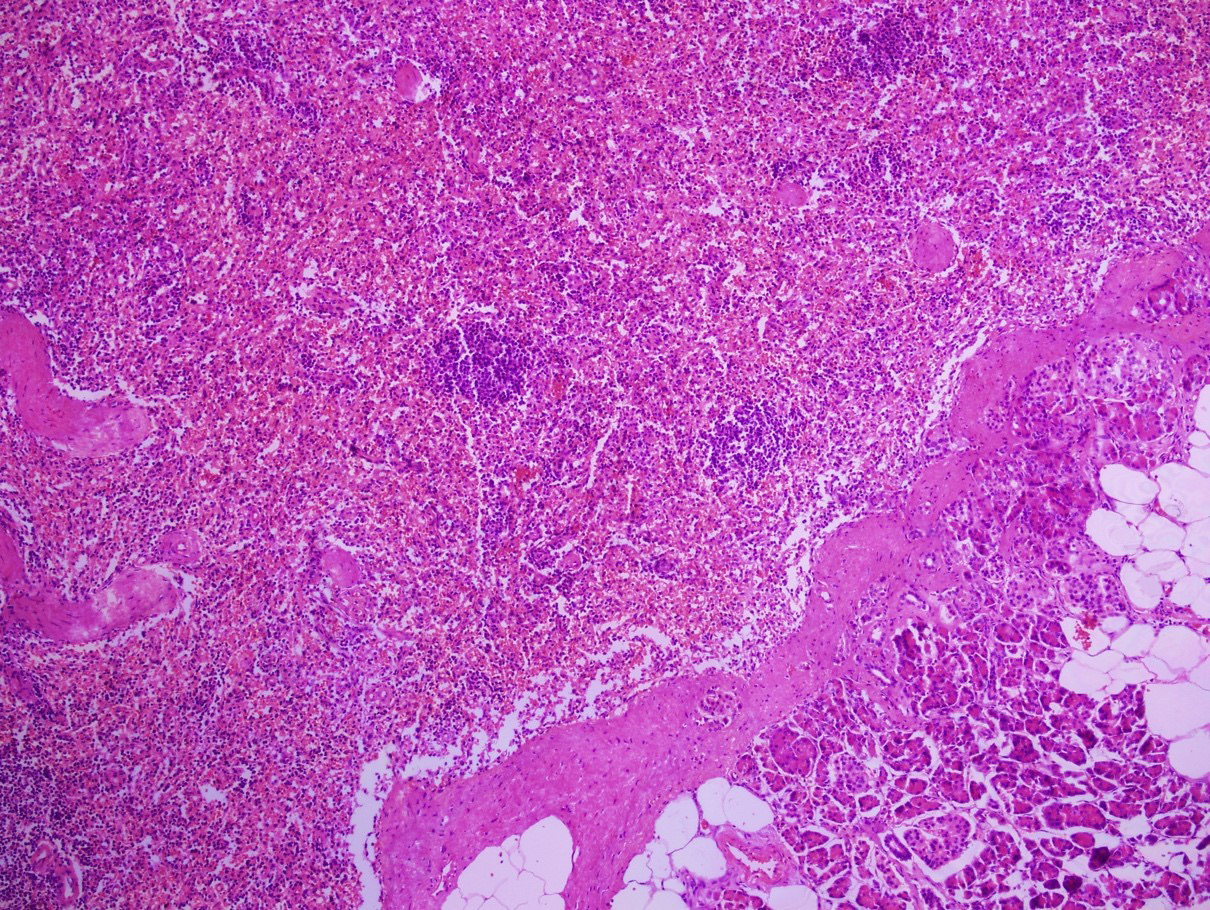
Intrapancreatic Accessory Spleen Tissue (Upper Left) Separated from the Pancreatic Tissue (Lower Right) by a Thin Capsule Structure (H&E, 10x)

## Discussion

 IPAS results from failure of splenic primordial buds to fuse during the embryological period.^5^ As it is clinically asymptomatic, it is usually seen as an incidental pancreatic mass in the patient who is investigated for gastrointestinal symptoms. Radiologically, they usually present as a well-defined, hypervascular and solid lesion located in the pancreatic tail; but this image is not characteristic for IPAS.^6,7^ Therefore, differential diagnosis should be made in terms of neuroendocrine tumors, solid pseudopapillary tumors, mucinous cystic neoplasms (MCNs), pancreatic adenocarcinomas and well-vascularized metastases (such as kidney carcinomas) which are hypervascular neoplastic lesions located in the pancreatic tail.^4,8^

 MCNs of the pancreas are one of the neoplasms located in the pancreatic tail and they are a disparate group of tumors which range from benign, borderline to malignant, and also predominate in women.^9^ MCNs are slow-growing tumors, so on long-period follow-up, they can mimic IPAS.^10^ MCNs are well-encapsulated, mostly round lesions which are located in the body and tail of the pancreas in 70%–90% of cases. Therefore, these tumors can be distinguished from IPAS due to their radiologically typical cystic appearance.^11^

 Solid pseudopapillary tumors are low-grade malignant neoplasm of uncertain cellular differentiation which occur predominantly in younger females. These tumors can be located in the head, body or tail of the pancreas.^12^ When they are located in the pancreatic tail, they can mimic IPAS, because they constitute a macroscopically well-demarcated, solitary mass. However, they are larger in size (the mean diameter 9–10 cm) than IPAS and they are differentiated radiologically as heterogeneous masses with solid cystic components.^13^

 Pancreatic ductal adenocarcinomas usually occur in the head of the pancreas. Only a minority of the cases occur in the tail of the pancreas and also 10%–20% of cases are resectable at diagnosis.^14^ Most of the time, diagnosis cannot be established by fine-needle aspiration biopsy due to difficulties arising from the anatomic location of the pancreas. Therefore, abdominal imaging has gained importance in pancreatic masses.^15^ For resectable tumors, surgery offers a chance of cure. As a result, when a mass is detected in the pancreas, pancreatomy surgery is acceptable for early-stage cancer because of the possibility of curative resection.

 Well-differentiated neuroendocrine tumor (WD-NET) of the pancreas is the most frequently confused entity with IPAS.^16^ WD-NETs usually appear as a solid mass that can arise in any portion of the pancreas. WD-NETs are divided into hormonally active and inactive neoplasms. Hormonally active WD-NETs such as insulinoma and gastrinoma are clinically symptomatic; so it is simpler to distinguish them from IPAS. However, most pancreatic WD-NETs are hormonally inactive, asymptomatic, and incidentally detected by imaging, similar to IPAS.^17^ These neoplasms are usually intraparenchymal and are typically best seen on computer tomography (CT) in the arterial phase of enhancement.^18^

 Accessory spleens can be easily recognized by their characteristic location and spleen-like appearance on CT and MRI.^19^ So, when a mass is detected in the pancreatic tail, the diagnosis of IPAS can be made radiologically by comparing the CT and MRI findings with the spleen enhancement findings.^20^ Nevertheless, it is sometimes difficult to distinguish IPAS from hypervascular pancreatic tumors radiologically, as they may have a similar radiologic pattern. When CT and MRI findings are inconclusive, different radiological imaging techniques may be needed to distinguish IPAS from other pancreatic tumors, such as heat-damaged red blood cell 99mTc scintigraphy, superparamagnetic iron oxide-enhanced MRI and contrast enhanced ultrasound.^21,22^

 When a mass is detected in the pancreas tail, if the diagnosis of IPAS is suspected, multimodal imaging may be helpful to confirm the diagnosis. Also, the lesion is more frequently aspirated under CT guide or fine-needle aspiration with endoscopic ultrasound guide to help the diagnosis. So, the patient may be protected from surgery for a benign condition. However, most of the cases of IPAS presented in the literature have been confirmed after surgery.

 We wish to remind that IPAS, which can mimic the pancreatic neoplasms clinically and radiologically, is a benign entity that should be kept in mind to avoid unnecessary surgery for patients.
